# Electrical stimulation of the common peroneal nerve and its effects on the relationship between corticomuscular coherence and motor control in healthy adults

**DOI:** 10.1186/s12868-021-00665-w

**Published:** 2021-10-13

**Authors:** Tadaki Koseki, Daisuke Kudo, Natsuki Katagiri, Shigehiro Nanba, Mitsuhiro Nito, Shigeo Tanabe, Tomofumi Yamaguchi

**Affiliations:** 1 Department of Physical Therapy, Yamagata Prefectural University of Health Sciences, 260 Kamiyanagi, Yamagata, 990-2212 Japan; 2 Department of Anatomy and Structural Science, Yamagata University School of Medicine, 2-2-2 Iida-Nishi, Yamagata, 990-9585 Japan; 3 Faculty of Rehabilitation, School of Health Sciences, Fujita Health University, 1-98 Dengakugakubo, Kutsukake-cho, Toyoake, Aichi 470-1192 Japan; 4 Department of Physical Therapy, Faculty of Health Science, Juntendo University, 2-1-1 Hongo, Bunkyo-ku, Tokyo, 113-8421 Japan

**Keywords:** Neuromuscular electrical stimulation, Beta-band stimulation, Sensory input, Corticomuscular coherence, Isometric contraction, Corticospinal excitability

## Abstract

**Background:**

Sensory input via neuromuscular electrical stimulation (NMES) may contribute to synchronization between motor cortex and spinal motor neurons and motor performance improvement in healthy adults and stroke patients. However, the optimal NMES parameters used to enhance physiological activity and motor performance remain unclear. In this study, we focused on sensory feedback induced by a beta-band frequency NMES (β-NMES) based on corticomuscular coherence (CMC) and investigated the effects of β-NMES on CMC and steady-state of isometric ankle dorsiflexion in healthy volunteers. Twenty-four participants received β-NMES at the peak beta-band CMC or fixed NMES (f-NMES) at 100 Hz on different days. NMES was applied to the right part of the common peroneal nerve for 20 min. The stimulation intensity was 95% of the motor threshold with a pulse width of 1 ms. The beta-band CMC and the coefficient of variation of force (Force CV) were assessed during isometric ankle dorsiflexion for 2 min. In the complementary experiment, we applied β-NMES to 14 participants and assessed beta-band CMC and motor evoked potentials (MEPs) with transcranial magnetic stimulation.

**Results:**

No significant changes in the means of beta-band CMC, Force CV, and MEPs were observed before and after NMES conditions. Changes in beta-band CMC were correlated to (a) changes in Force CV immediately, at 10 min, and at 20 min after β-NMES (all cases, p < 0.05) and (b) changes in MEPs immediately after β-NMES (p = 0.01). No correlations were found after f-NMES.

**Conclusions:**

Our results suggest that the sensory input via NMES was inadequate to change the beta-band CMC, corticospinal excitability, and voluntary motor output. Whereas, the β-NMES affects the relationship between changes in beta-band CMC, Force CV, and MEPs. These findings may provide the information to develop NMES parameters for neurorehabilitation in patients with motor dysfunction.

## Introduction

Motor control is essential for the interactions with the environment. It is heavily dependent on the descending cortical control of the muscles [1, 2]. Specifically, the developed steady-state forces during daily living (i.e., grasping a cup, standing) are controlled by sensorimotor binding through somatosensory feedback to cortex [3]. Therefore, the enhancement of the communication between cortex and muscles may improve motor control that may in turn contribute to the development of rehabilitation strategies for patients who suffer from motor dysfunction.

Corticomuscular coherence (CMC) is a method used to assess synchronization between motor cortex and spinal motor neurons activities [1, 3–8]. The magnitude of beta-band CMC (15–35 Hz) is correlated with the ability to stabilize muscle force output [3]. The beta-band CMC is significantly lower in stroke patients compared with healthy controls [9, 10]. Alternatively, the enhancement in beta-band CMC is related to motor learning processes [7, 11], and motor functional recovery after stroke [12]. Thus, the development of a technique to increase beta-band CMC is expected to enhance motor learning and promote motor recovery in patients with stroke.

Several studies have demonstrated that beta-band CMC is modulated by an afferent input [6, 13–16]. One possible strategy for the enhancement of CMC is neuromuscular electrical stimulation (NMES). The peripheral sensory inputs via NMES enhanced the activities of circuits in the somatosensory cortex [17–19]. The activation of the somatosensory cortex by NMES indirectly enhances the excitability of primary motor cortex and projections to the spinal cord [19–21]. Therefore, NMES enhances beta-band CMC and motor functional recovery in individuals with stroke [22]. However, another study has reported that NMES enhances gamma-band CMC, but beta-band CMC did not significantly change [23]. These results suggested that the sensory input via NMES may affect CMC and motor performance, although the evidence has been inconclusive. Additionally, prior studies only used fixed NMES frequencies (e.g., 100 Hz). To the best of our knowledge, no prior study has focused on the beta-band frequency of NMES based on physiological activity, and the mechanisms underlying these effects still remain unclear.

A previous study reported that transcranial alternating current stimulation (tACS) with stimulus frequency based on individual’s beta-band CMC enhances CMC and performance retention [24]. Therefore, we hypothesized that NMES with beta-band CMC frequency changes CMC and muscle force control. In the present study, we investigated whether NMES corresponding to beta-band CMC frequency can enhance synchronization between motor cortex and spinal motor neurons, and whether it can improve the exerted steady-state of ankle dorsiflexion in healthy individuals.

## Methods

### Participants

Twenty-four healthy volunteers (12 females) aged 21–30 years old, (mean ± standard deviation: SD, 22 ± 2 years) participated in the experiment. None of the participants had any history of neurological and/or musculoskeletal disorders. The number of participants was decided based on a previous study that investigated the modulation of CMC by NMES [23]. All participants were right-foot dominant as confirmed by the foot-dominant test [25]. All participants provided written informed consent before participation. The experimental protocol was approved by the Ethical Review Board of Yamagata Prefectural University of Health Science in Japan and conformed to the ethical standards laid down in the 1964 Declaration of Helsinki.

### NMES

NMES was delivered with a stimulator (SEN-3401, Nihon Kohden, Tokyo, Japan). The first stimulation frequency for NMES was defined as beta-band frequency NMES (β-NMES) and was estimated from the peak of the beta-band CMC frequency (mean ± SD, 21 ± 5 Hz), while participants performed isometric dorsiflexion tasks before the stimulation. The second stimulation frequency for NMES was defined as the fixed frequency of NMES (f-NMES) and consisted of a train of 10 pulses at 100 Hz generated every 2 s [26]. The electrodes were applied to the skin above the right common peroneal nerve (CPN) for 20 min [27]. The stimulation intensity was 95% of the motor threshold with a pulse width of 1 ms. The motor threshold was defined such that the minimum stimulation intensity evoked muscle twitch contraction of the tibialis anterior (TA) muscle.

### Electroencephalographic (EEG) and electromyographic (EMG) recordings

The participants sat comfortably on a chair with their feet firmly strapped to a foot plate. EEG and EMG were recorded with Ag/AgCl electrodes. Before the attachment of electrodes, the skin was rubbed with an alcohol pad and the skin impedance was kept below 5 kΩ. EEG electrodes were placed at Cz representing the ankle muscles and at 5 cm frontal to Cz, according to the international 10–20 system of electrode placement [7]. EMG electrodes were placed over the TA muscle belly in the right lower limb with an inter-electrode distance of 2 cm. EEG and EMG data were simultaneously acquired (Neuropack MEB-2200, Nihon Kohden, Tokyo, Japan), bandpass filtered in the frequency range of 0.5–200 Hz in the case of EEG and in the range of 5–500 Hz in the case of EMG. Force signals were recorded with a force transducer (Takei Scientific Instruments Co. Niigata, Japan). EEG, EMG, and force data were recorded while the participants performed isometric dorsiflexion to maintain their exerted force as close as possible to the line that corresponded to 10% of their maximum voluntary contraction (MVC) force for 2 min [28]. All signals were converted to digital signals at a sample frequency of 5 kHz by analog-to-digital converter with a 16-bit resolution (NI USB-6363, National Instruments, Austin, TX, USA) controlled by the data-logger software LabVIEW2018 (National Instruments Co., TX, USA).

### Corticomuscular coherence

CMC was estimated by EEG and EMG data following Eq. (1) [29]. CMC describes the linear association between EEG and EMG signals at each frequency of interest. It is a measure of phase consistency between signals. CMC estimates are defined over the range 0–1, where the value of zero indicates no association, and one indicates a perfect association. To calculate CMC, auto-spectra and cross-spectra were constructed by dividing signals into nonoverlapping segments. Discrete Fourier transforms were then performed on each segment and averaged. CMC was then determined as the squared modulus of the cross-spectrum for the two signals f_xy_(j), normalized by the product of the two auto-spectra, f_xx_(j), and f_yy_(j) [28].

1 $$\left| {Rxy\left( j \right)} \right|^{2} = \frac{{\left| {fxy\left( j \right)} \right|^{2} }}{fxx\left( j \right)fyy\left( j \right)}$$

The statistical significance of CMC estimates was assessed according to an upper 95% confidence limit.

### Force steadiness of ankle dorsiflexion

The right foot was strapped to the plate with the ankle in the neutral position. Participants were asked to maintain an isometric dorsiflexion at 10% MVC for 2 min. The target force line was presented in front of participants with a display. Participants were instructed to follow the target line as closely as possible with the moving red line (real-time dorsiflexion force). It was reported that the beta-band CMC was negatively correlated with the coefficient of variation [(standard deviation/mean) × 100%] of force (Force CV)^3^. If NMES changes the beta-band CMC, the changes in the beta-band CMC could be related with the changes in the exerted steady-state of ankle dorsiflexion. To investigate the exerted steadiness of dorsiflexion force during the ankle dorsiflexion task, Force CV was calculated as a measure of the control of the exerted force.

### Experimental procedure

**Fig. 1:**
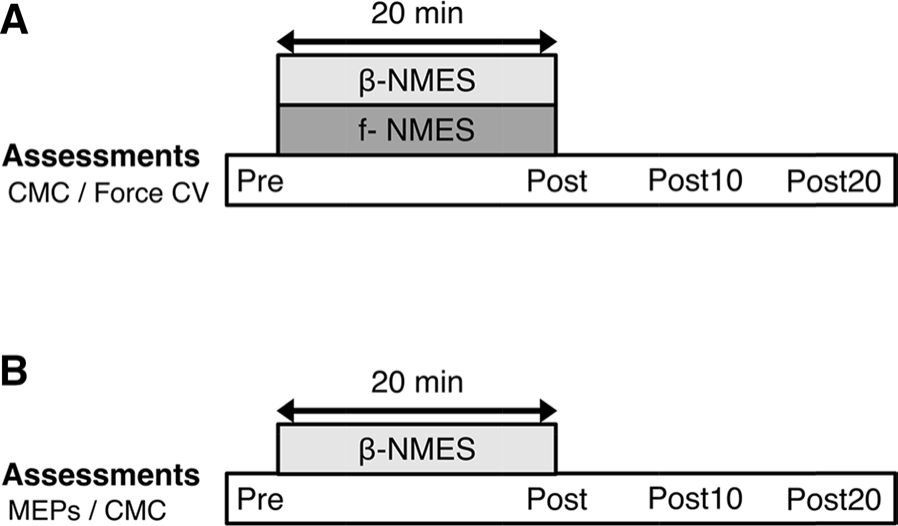
Experimental paradigm. Time course of main experimental (**A**) and Complementary experiment (**B**). In the main experiment, all participants received neuromuscular electrical stimulation (NMES) at two different frequencies as follows: (1) beta-band frequency NMES (β-NMES) frequency estimated by the peak of the beta-band corticomuscular coherence (CMC) in the range of 15–35 Hz and (2) a fixed 100 Hz NMES (f-NMES) frequency. The beta-band CMC and the coefficient of variation of force (Force CV) were assessed before stimulation (pre) and after stimulation at 0 min (post), 10 min (post10), and 20 min (post20). In the complementary experiment, all participants received β-NMES. The motor evoked potentials (MEPs) and the beta-band CMC were assessed before stimulation (pre) and after stimulation at 0 min (post), 10 min (post10), and 20 min (post20)

This study employed a randomized crossover design. Participants received β-NMES and f-NMES on two different days (Fig. 1A). To assess beta-band CMC changes, EEG and EMG were measured before stimulation (pre) and after stimulation at 0 min (post), 10 min (post10) and 20 min (post20). To prevent carry-over effects from the previous NMES condition, washout intervals of 3 days (or more) were included between sessions.

### Complementary experiment

Beta-band CMC reflects the functional coupling between primary motor cortex and spinal motor neurons that related to corticospinal excitability [9, 30]. Corticospinal excitability was modulated by NMES [27, 31–33]. Therefore, the changes in the beta-band CMC induced by β-NMES may be related to the changes in corticospinal excitability. To address this question, 14 healthy volunteers (5 females) aged 20–32 years old, (mean ± SD, 24 ± 3 years) received β-NMES (mean ± SD, 25 ± 5 Hz) for 20 min (Fig. 1B). To assess corticospinal excitability, single-pulse transcranial magnetic stimulation was delivered to the primary motor cortex responsible for motor representation of the leg with a figure-8 coil (D70^2^) that was connected to the Magstim 200 (Magstim Company, Whitland, United Kingdom). The optimal coil positioning on the hot spot of the primary motor cortex was identified for the induction of the largest motor evoked potentials (MEPs) amplitudes in the right TA. The stimulation intensity was adjusted to 120% of the active motor threshold (aMT). The aMT was defined as the minimum stimulus intensity that produced 200 μV MEPs with a probability of 50% during isometric contraction at 100 μV of TA EMG. Fifteen MEPs were recorded while the participants performed an isometric contraction with 100 μV of TA. Before the main assessment, baseline MEPs and CMC were measured to normalize the data. Following a 2-min rest period, MEPs and CMC were assessed pre, post at 0 min (post), 10 min (post10), and 20 min (post20). To minimize the influences of voluntary contraction during the CMC assessment, the MEPs were assessed experimentally before the CMC assessment.

### Statistical analysis

Normality was assessed with the Shapiro–Wilk test. The Wilcoxon signed-rank test was used to compare pre data between the raw value of the beta-band CMC and Force CV. Friedman test was used to investigate the main effect of time (pre, post, post10, and post20) on the raw values of the beta-band CMC and Force CV. Post-hoc tests were performed using the Wilcoxon signed-rank test with Bonferroni adjustments when a significant main effect was found. Spearman’s rank correlation was used to define the relationship between the normalized beta-band CMC and the Force CV induced by stimulations in assessment time points (post, post10, and post20). The normalized beta-band CMC and normalized Force CV were calculated by the obtained data pre.

In the complementary experiment, the normalized beta-band CMC and normalized MEPs were calculated with the baseline data. Friedman’s test was used to investigate the main effect of time (pre, post, post10, and post20) on the normalized MEPs. Post-hoc tests were performed with the Wilcoxon signed-rank test with Bonferroni adjustments when a significant main effect was found. Pearson’s product moment correlation was used to investigate the relationship between the normalized MEPs and normalized beta-band CMC at assessment time points after β-NMES (pre, post, post10, and post20). P-values < 0.05 indicated statistical significance in all analyses. Statistical analyses were performed with SPSS (version 24.0, IBM Corporation, New York, NY, USA) for Windows.

## Results

The Shapiro–Wilk test showed that the main experimental data on the beta-band CMC, Force CV, normalized beta-band CMC, and normalized Force CV were not normally distributed.

### CMC

**Fig. 2:**
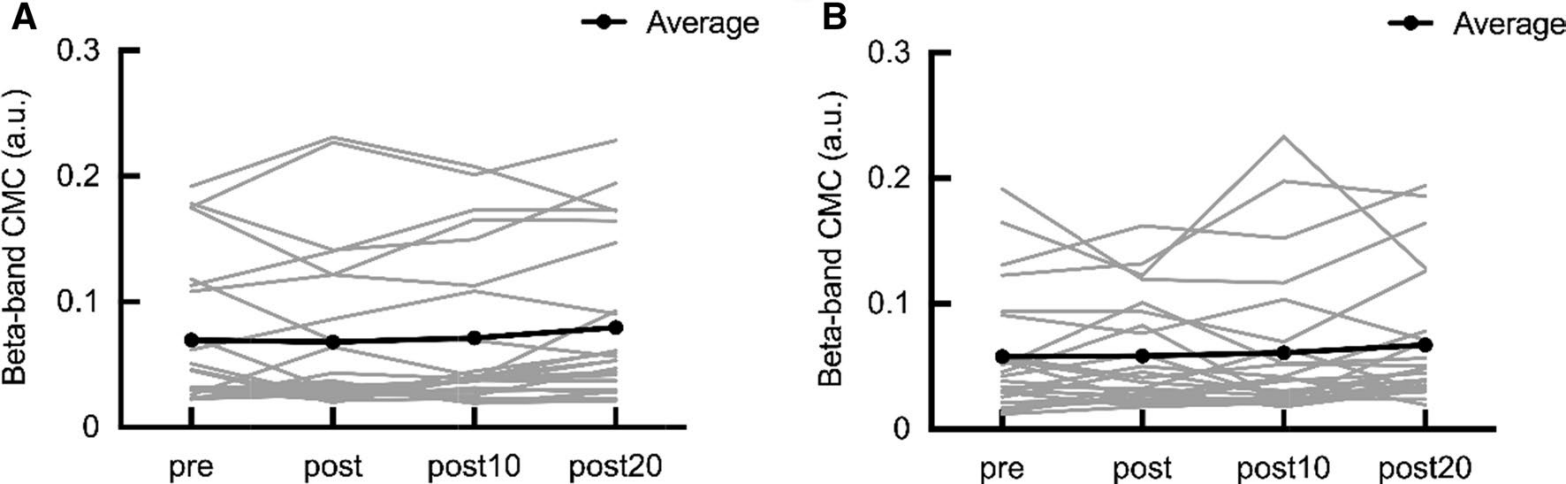
Effects of NMES on beta-band CMC. The gray lines represent the individual participant data of beta-band CMC. The black line represents average data. **A** Effects of the β-NMES frequency on the beta-band CMC. **B** Effects of the f-NMES frequency on the beta-band CMC

The mean raw data (SD) of the beta-band CMC at pre were 0.07 (0.06) in β-NMES and 0.06 (0.05) in f-NMES. No significant difference was observed between β-NMES and f-NMES conditions at pre (Wilcoxon signed-rank test, p = 0.08). No significant main effects were observed on β-NMES (p = 0.21) (Fig. 2A) and f-NMES (p = 0.11) (Fig. 2B).

### Force CV

The mean raw data (SD) of the Force CV at pre were 2.9 (1.3) % in β-NMES and 2.7 (1.0) % in f-NMES. No significant difference was observed between β-NMES and f-NMES conditions at pre (Wilcoxon signed-rank test, p = 0.22). Furthermore, no significant main effects were found on β-NMES (p = 0.13) and f-NMES (p = 0.74).

### Relationship between normalized beta-band CMC and Force CV

**Fig. 3:**
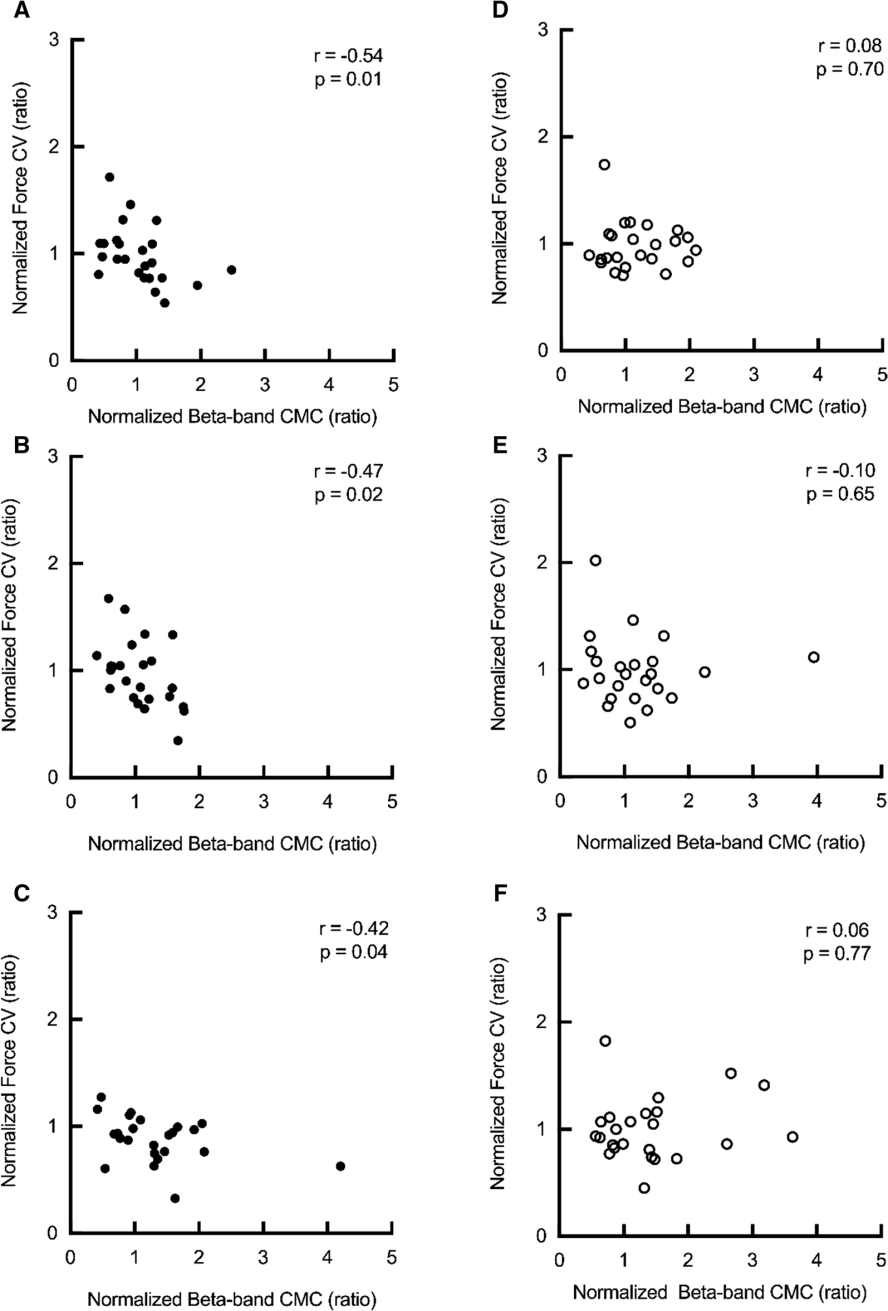
Correlations between normalized beta-band CMC and normalized Force CV at each assessment time point. Data plots represent the relationships between the normalized beta-band CMC and normalized Force CV on the β-NMES (closed circle) at post (**A**), at post10 (**B**), and at post20 (**C**). Data plots represent that relationships between the normalized beta-band CMC and normalized Force CV on f-NMES (opened circle) at post (**D**), at post10 (**E**), and at post20 (**F**)

Figure 3 shows data plots on the normalized beta-band CMC and normalized Force CV. For β-NMES condition, there were significant negative correlations at post (r =  − 0.54, p = 0.01, Fig. 3A), post10 (r =  − 0.47, p = 0.02, Fig. 3B), and post20 (r =  − 0.42, p = 0.04, Fig. 3C). However, the f-NMES condition showed that no significant correlations were found at post (r = 0.08, p = 0.70, Fig. 3D), post10 (r =  − 0.10, p = 0.65, Fig. 3E), and post20 (r = 0.06, p = 0.77, Fig. 3F). These results indicate that changes in beta-band CMC following β-NMES are related with the changes in the exerted steady state of ankle dorsiflexion, meaning that the relationship is modulated by NMES with a beta-band CMC frequency.

### Complementary experiment

#### MEPs

**Fig. 4:**
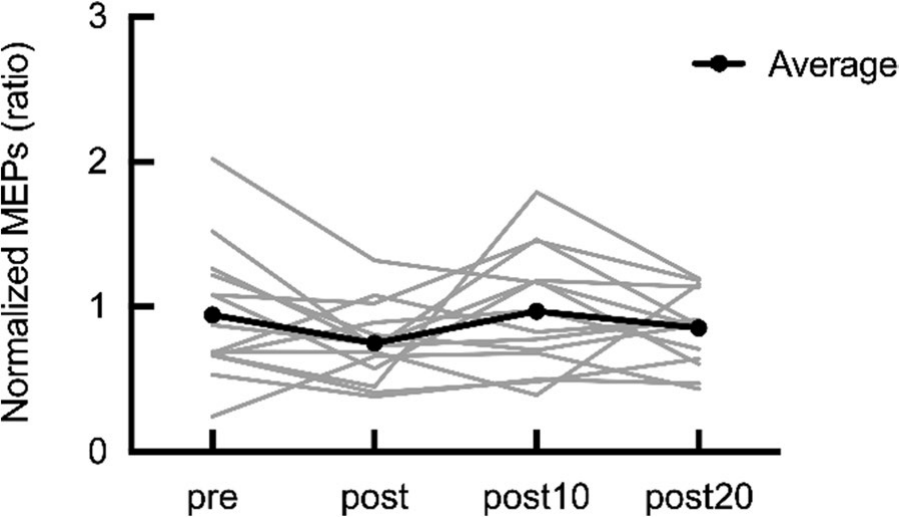
Effects of the β-NMES on the motor evoked potentials (MEPs) of the tibialis anterior muscle. The gray lines represent the individual participant data with normalized MEP amplitude. The black line represents average data

A significant main effect was observed on the normalized MEPs (p = 0.02). However, post-hoc analysis revealed no significant differences at each time point compared with data pre (p > 0.05, Fig. 4).

### Relationship between the normalized MEPs and normalized beta-band CMC

**Fig. 5:**
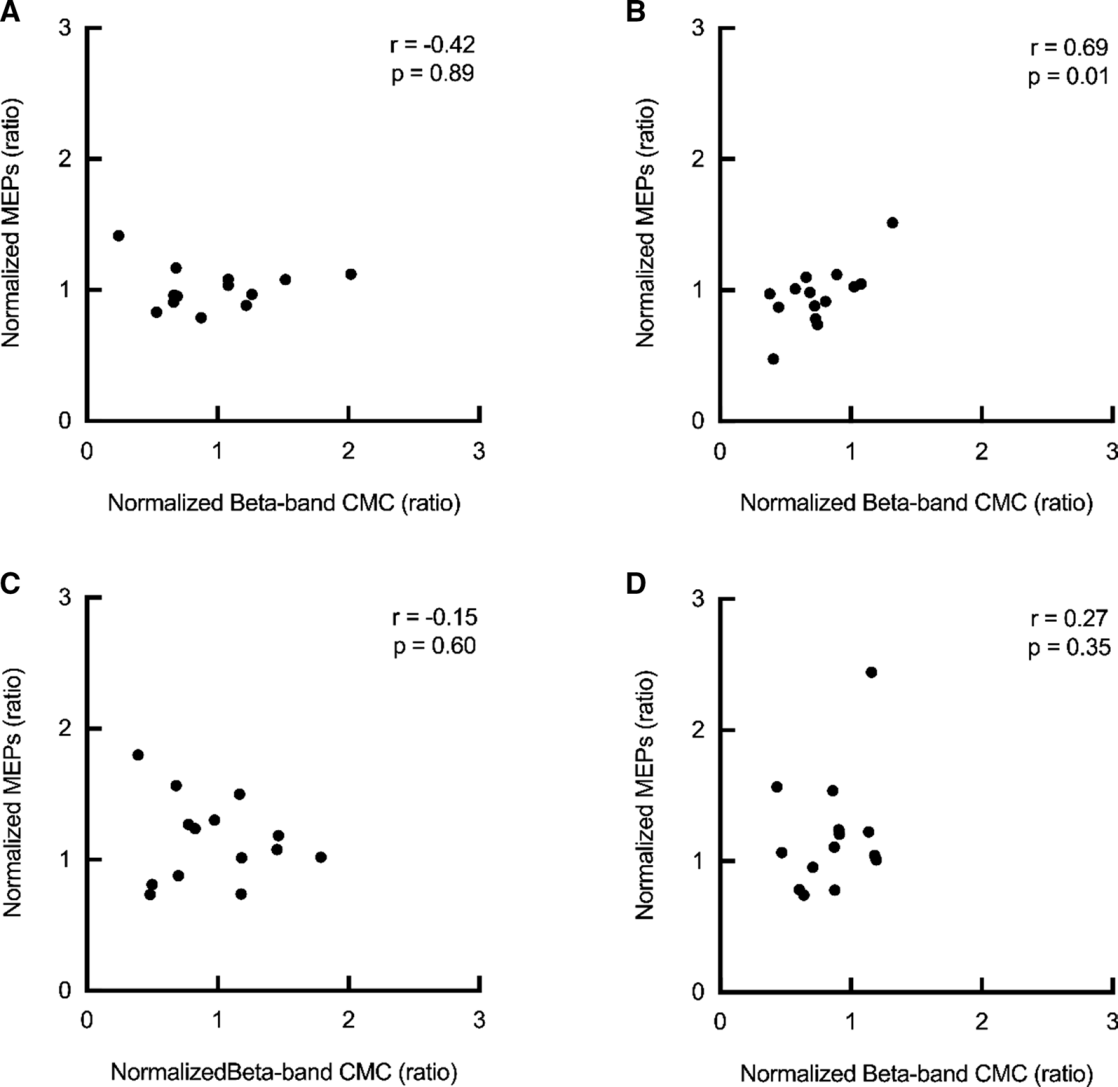
Correlations between the normalized beta-band CMC and normalized MEPs at each assessment time point. Data plots represent that relationships between the normalized beta-band CMC and normalized MEPs on β-NMES at pre (**A**), post (**B**), post10 (**C**), and post20 (**D**)

Figure 5 shows data plots on the normalized MEPs and normalized beta-band CMC. There was a significant positive correlation at post (r = 0.69, p = 0.01, Fig. 5B), whereas no significant correlations were observed at pre (r =  − 0.42, p = 0.89, Fig. 5A), at post10 (r =  − 0.15, p = 0.60, Fig. 5C), and at post20 (r = 0.27, p = 0.35, Fig. 5D). The results indicated that changes in corticospinal excitability were related with data in beta-band CMC following β-NMES.

## Discussion

The present study demonstrated no significant changes in the mean data of beta-band CMC, Force CV, and normalized MEPs in both the β-NMES and f-NMES cases. Whereas, NMES with beta-band CMC frequency affects changes in synchronization between the motor cortex and spinal motor neurons that correlate with changes in the exerted steady-state of isometric ankle dorsiflexion and changes in corticospinal excitability. These results may lead to the understanding of the mechanism underlying the sensory input via the beta-band frequency NMES based on physiological activity effects on corticospinal excitability and voluntary motor output. This suggestion may contribute to the development of effective NMES parameters for a neurorehabilitation in patients with motor dysfunction.

We expected that the β-NMES enhances the beta-band CMC, Force CV, and normalized MEPs, but the results were unexpected. In the present study, β-NMES enhanced the beta-band CMC in 13 of the 24 participants (54%). One possibility for these unexpected results is that there is variability of the effects of β-NMES on CMC [22, 23, 34–36]. A review paper regarding NMES parameters suggested that the intervention duration and stimulation intensity of NMES are important parameters for the modulation of the corticospinal excitability [34]. We set the duration of NMES to 20 min based on a previous study that has reported that the MEPs increased following 20 min of NMES [27]. Further consideration of the fact that the CMC changes were induced by muscle fatigue [37, 38], NMES with 95% of the motor threshold was set in this study. A study has reported that NMES with an intensity of below the motor threshold activated the cutaneous and sensory fibers and increased corticospinal excitabilities [39, 40]. However, 20-min NMES with an intensity of below the motor threshold might be insufficient to affect CMC and corticospinal excitability in this study. Indeed, a previous study has reported that an intensity below the motor threshold and/or a frequency of 100 Hz do not enhance the beta-band CMC [23]. Therefore, future research is warranted to investigate the effects of NMES with different intervention durations and stimulation intensities on CMC.

Conversely, the correlation between changes in beta-band CMC and changes in Force CV were observed continually at least 20 min. Previous studies reported that the concentration of cortical oscillation to specific frequency (e.g., 20 Hz) reduces the variability of common synaptic inputs, and motor units discharge and improve the exerted steady-state force [41–43]. Thus, changes in the exerted steady-state of isometric ankle dorsiflexion are affected by changes in control from the motor cortex to spinal motor neurons, that is, by changes in the synchronization between them. The changes in beta-band CMC reflected changes in descending drive from the primary motor cortex to spinal motor neurons. This is supported by our complementary experiment indicating the correlation between changes in beta-band CMC and changes in MEPs immediately after β-NMES. Another study reported that cortical oscillation may modulate the firing rate of motor cortical efferent commands [1]. The cortical oscillation desynchronizes immediately after the afferent input via the electrical stimulation, and then rebounds to a more effective synchronization than before stimulus [44]. Thus, our results suggested that β-NMES alters the corticospinal projections by inducing changes in the degree of interneuron’s synchronization within the primary motor cortex. These physiological changes may affect the steady state of isometric ankle dorsiflexion. Alternatively, the correlation between changes in beta-band CMC and those in Force CV was weak. Studies have reported that beta-band CMC was influenced by muscle fatigue and attention [37, 38, 45, 46]. These factors might affect the results of this study [47].

In contrast, the correlation between changes in beta-band CMC and changes in Force CV was not observed after f-NMES at a frequency of 100 Hz. The f-NMES was set to emulate burst stimulation based on the sensory feedback from TA muscle spindles induced owing to the synaptic plasticity of the spinal interneuron circuit [26]. However, no changes were observed in corticospinal excitability in their study. Our complementary experiment observed the correlation between changes in beta-band CMC and changes in MEPs. Thus, the changes in the corticospinal excitability were responsible for the correlation between changes in beta-band CMC and changes in Force CV and not the plasticity in spinal interneuron.

The effects of fixed frequency within the beta-band (i.e., 20 Hz) of NMES were not investigated in the present study. Additional studies are needed to (a) investigate the effect of fixed frequency within the beta-band of NMES, and (b) identify more effective stimulation time and intensity to enhance the mean data on beta-band CMC, Force CV, and MEPs. This study was conducted on healthy participants. Future studies are needed to test the current approach in patients with sensorimotor dysfunction.

## Conclusion

The present study shows that NMES with beta-band CMC frequency was inadequate to enhance CMC, Force CV, and MEPs. However, β-NMES affects the relationship between changes in beta-band CMC, Force CV, and MEPs. We proposed that sensory input based on individual beta-band CMC frequencies may affect the relationship between beta-band physiological activity and voluntary motor output. Additional experiments are required to investigate the influence of the intervention duration and stimulation intensity.

## Data Availability

The datasets used and/or analyzed in the current study are available from the corresponding author on reasonable request.

## Abbreviations

aMT: Active motor threshold
β-NMES: Beta-band frequency neuromuscular electrical stimulation
CPN: Common peroneal nerve
CMC: Corticomuscular coherence
EEG: Electroencephalographic
EMG: Electromyographic
Force CV: Coefficient of variation of force
f-NMES: Fixed frequency neuromuscular electrical stimulation
MEPs: Motor evoked potentials
MVC: Maximum voluntary contraction
NMES: Neuromuscular electrical stimulation
TA: Tibialis anterior
tACS: Transcranial alternating current stimulation
